# Clinical study of the Pronator Quadratus muscle: anatomical features and feasibility of Pronator-Sparing Surgery

**DOI:** 10.1186/1471-2474-15-136

**Published:** 2014-04-24

**Authors:** Hsien-Yi Lo, Hong-Yi Cheng

**Affiliations:** 1 Institute of Neuroscience, National Chengchi University, NO.64, Sec.2, ZhiNan Rd., Wenshan District, Taipei City 11605, Taiwan; 2 Department of Orthopaedic Surgery, Puzi Hospital, Ministry of Health and Welfare, Chiayi City 61347, Taiwan; 3 Department of Orthopaedic Surgery, Yangming Branch, Taipei City Hospital, Taipei City 11146, Taiwan

**Keywords:** Pronator quadratus muscle, Distal radius fracture, Pronator-sparing approach

## Abstract

**Background:**

No clinical data for the relationship of pronator quadratus (PQ) muscle to distal radius had been reported. The aim of this study was to investigate the anatomical features of the PQ muscle related to plate osteosynthesis for distal radius fractures in clinical cases. The feasibility of PQ muscle sparing surgery was investigated as well.

**Methods:**

Fifty two distal radius fractures (23-A2) were enclosed in this study. The whole width of the muscle and the distance from the distal edge of the muscle to the joint surface of the distal radius were measured. The distance from the fracture site of the radius to the joint surface was measured as well.

**Results:**

The average width of the pronator quadratus muscle was 37.6 mm. The average distance from the pronator quadratus muscle to the lunate fossa surface was 12.2 mm, and the average distance from the pronator quadratus muscle to the scaphoid fossa surface was 13.6 mm. The average distance from the lunate fossa of the distal radius to the fracture site was 12.2 mm (range, 7.3-17 mm), and the scaphoid fossa of the distal radius to the fracture site was 13.2 mm (range, 9.4-18.8 mm).

**Conclusions:**

This PQ muscle sparing surgery is feasible and can be performed without difficulty. The data might provide a useful basis for the preservation of pronator quadratus muscle applied to a functionally reduced fracture regarding the potential efficacy of conventional volar plate osteosynthesis.

## Background

The traditional method of open reduction and plate fixation for distal radius requires wide exposure of the fracture site with stripping of the soft tissues which may devascularize the fracture fragments [1]. This will contribute to the necrosis caused by trauma itself or operation and, consequently, increase the risks of delayed healing and infection. Minimally invasive plate osteosynthesis (MIPO) was developed to avoid wide exposures of the fracture site and minimize soft tissue damage [2]. It has been used most frequently for fixation of lower extremity fractures [3,4]. More recently, its use has been described for upper extremity fractures [5]. The advantages of MIPO are that it causes less damage to the soft tissue and preserves periosteal circulation in the fractured fragments. Imatani et al. [6]. described MIPO of distal radial fractures through two small skin incisions without division of the pronator quadratus (PQ) muscle. This concept was expanded in our conventional approach and plate fixation for distal radius fracture.

The anatomic pictures of the PQ muscle in a cadaver study was demonstrated by Takada and Otsuka [7]. There was no live report related to fracture site of the distal radius to the joint surface and PQ muscle yet. We presented a clinical study to show the anatomic features of the PQ muscle during open surgical reduction for distal radius. The feasibility of PQ muscle sparing in conventional surgery for distal radius fracture was also investigated.

This study was conducted prospectively and the aims of this work are:

1. To investigate the anatomical features of the pronator quadratus (PQ) muscle related to plate osteosynthesis for distal radius fractures in clinical cases.

2. The feasibility of PQ muscle sparing surgery was investigated as well.

## Methods

This study was conducted prospectively and approved by the Research Ethics Committee of the Taipei city hospital. Fifty two distal radius fractures were enclosed in this study between January 2009 and December 2011. According to the AO classification, the type of fracture of the radius in all cases was 23-A2. The FCR approach was used in this study. Namely, a longitudinal incision about 5 cm was made over the tendon of flexor carpi radialis (FCR). As the palmar cutaneous branch of the median nerve is almost always ulnar to the FCR tendon, the FCR tendon is then exposed by releasing its superficial sheath sharply. In addition, when releasing the sheath, one must be careful distally near the proximal wrist crease as the superficial branch of the radial artery crosses superficial to the sheath and runs from ulnar to radial. Upon releasing the FCR sheath, the FCR tendon is retracted radially. The deeper sheath and fascia under the FCR tendon are then released. At this point, dissection is done between the radial fascia and the FPL (flexor pollicis longus) muscle that runs deep to the fascia. The radial artery runs radial to the FCR tendon and superior to this fascia. Thus, when the radial fascia is retracted, the radial artery is protected. The FPL muscle must then be bluntly dissected and ulnar retraction be performed. The bed of the FCR tendon sheath was incised in line with the skin incision. Blunt dissection was then preformed to expose PQ.

A 21 gauge needle was used to identify the distal border of the scaphoid and lunate fossa of radius. A minimal capsulotomy was performed to make sure the position as the reference point of measurement. The fracture is distracted and the achieved closed reduction is checked by fluoroscopy. When a good reduction has been achieved it is temporarily stabilized with two 1.8 mm Kirschner wires driven into the radial styloid in a distal to proximal direction. The fracture site was usually around the distal edge of PQ muscle or covered by it.

**Figure 1:**
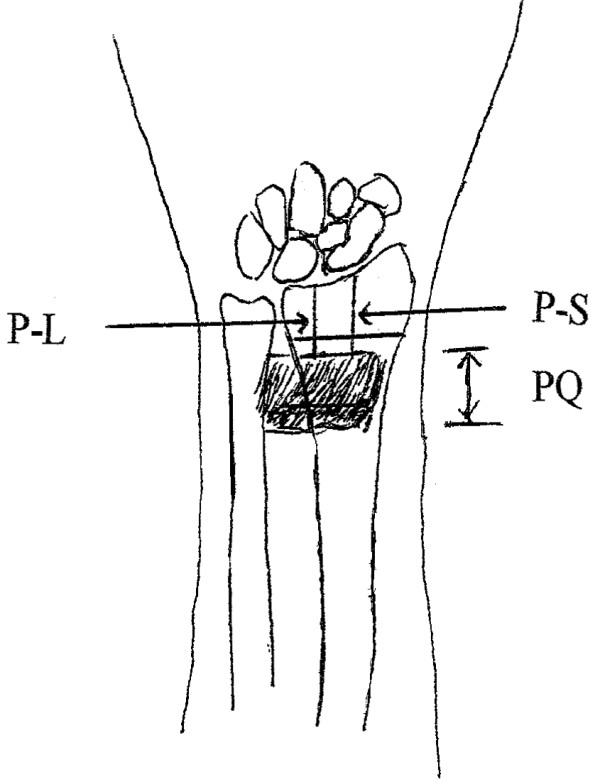
**A diagram demonstrated the relationship of distal radius and measurement of pronator quadratu, distance from distal edge of pronator quadratus to lunate or scaphoid fossa.** Likewise, the distance from fracture site to lunate or scaphoid fossa could also be measured. PQ: pronator quadratus muscle, P-L: distance from pronator quadratus to lunate fossa, P-S: distance from pronator quadratus to scaphoid fossa.

At this point the maximum width of PQ along the anatomical axis of the radius was measured. Measurements were also made from the distal margin of the radius in line with the lunate fossa and scaphoid fossa to the distal margin of the PQ. The distance from fracture site to lunate fossa and that to scaphoid fossa were also measured (Figure 1). All measurements were recorded along the surface of the bone in millimeters using a vernier caliper.

**Figure 2:**
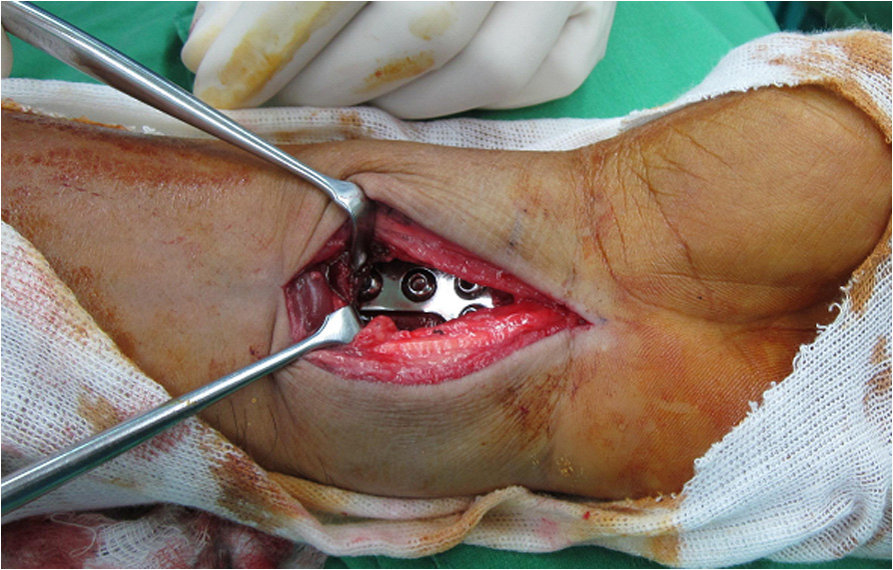
**The distal and middle holes of longitudinal limb were exposed by traction of PQ muscle.** The proximal screw hole could be placed under blunt dissection. The fracture site and joint line could be identified as well.

The space between PQ muscle and bone was created by an elevator and the plate was inserted beneath PQ muscle. Distal fixation was obtained first with one 3.5 mm screw inserted just beneath the subchondral bone at a convergent angle of 10° to the articular surface. Then, the longitudinal limb of the plate was lined up with the radial shaft, and the position of the plate was adjusted under fluoroscopy. The most proximal screw of the plate may be inserted through PQ under blunt dissection. The distal and middle screws of longitudinal limb of the plate were inserted easily with retraction of PQ muscle (shown in Figure 2). Other screws were then placed to the holes of the transverse limb. The reduction position was checked by routine radiogram postoperatively. Active motion of fingers and wrist are started at next day after surgery.

## Results

**Figure 3:**
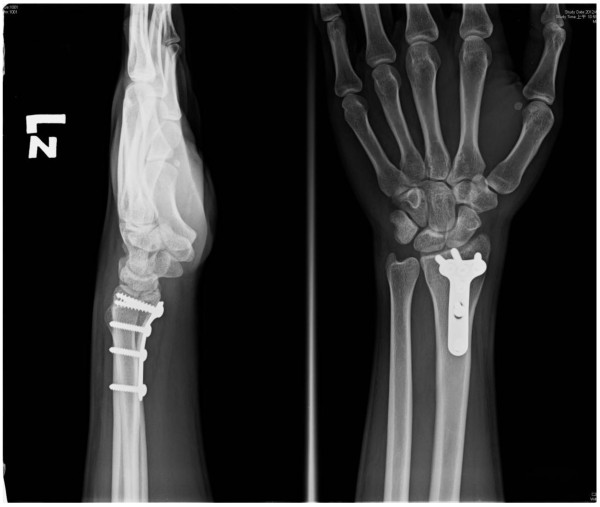
The Postoperative film shows good reduction of the fracture and position of the plate.

As Table 1 illustrated, the average width from the proximal edge to the distal edge of the PQ muscle was 37.2 mm (range, 30–41 mm). The average distance from the distal edge of the PQ muscle to the lunate fossa of the distal radius was 16.2 mm (range, 12–19 mm), and the average distance from the distal edge of the PQ muscle to the scaphoid fossa of the distal radius was 17.6 mm (range, 11–21 mm). The average distance from the fracture sites to the lunate fossa of the distal radiu was 12.2 mm (range, 7.3-17 mm), and the fracture site to the scaphoid fossa of the distal radius was 13.2 mm (range, 9.4-18.8 mm). There were 43 fractures located distal to the distal edge of PQ muscle. The Postoperative film (Figure 3) shows good reduction of the fracture and position of the plate.

**Table 1:** The width of pronator quadratus and distances between pronator quadratus or fracture site to the distal radial edge

	**Mean (mm)**	**Range (mm)**
PQ width	37.2	30-49
PQ-lunate	16.2	12-19
PQ-scaphoid	17.6	11-29
Fracture-lunate	12.2	7.3-17
Fracture-scaphoid	13.2	9.4-18.8

PQ: pronator quadratus; PQ-lunate:distance from pronator quadratus to lunate fossa; PQ-scaphoid: distance from pronator quadratus to scaphoid fossa; Fracture-lunate: distance from fracture site to lunate fossa; Fracture-scaphoid: distance from fracture site to scaphoid fossa.

## Discussion

Pronator quadratus (PQ) muscle is a quadrilateral muscle with attachments at the distal volar aspect of the ulna and radius [8]. Cadaver studies have confirmed the importance of the PQ muscle in the function of the forearm. Stuart [9] reported that the superficial head of the PQ muscle is the prime mover in forearm pronation and the deep head is a dynamic stabilizer of the distal radioulnar joint in a study of healthy volunteers. In this study, we did not try to identify these two portions. McConkey et al. [10]. showed that paralysis of the PQ muscle with lidocaine resulted in a significant decrease in pronation torque. The PQ muscle also has been shown to be essential to the blood supply of the distal radius, which is thought to be important in fracture healing. Rath et al. [11]. reported that the anterior interosseous artery supplies blood to the PQ muscle, and Lee et al. [12]. demonstrated that, in addition to the PQ, this artery also supplies blood to the distal radial periosteum. Incision of the PQ inevitably damages the blood supply. Tobe et al. [8]. suggested that the MIPO technique prevents soft tissue damage and adhesions around the PQ muscle and provides good functional results immediately after surgery. Sen and Harvey [13] described a “pronator-sparing” technique and cited several advantages: decreased stiffness, lower risk of postoperative tendon rupture because the PQ muscle serves as a protective layer between the plate and the flexor tendons, additional stability of the distal radioulnar joint, and preservation of the volar blood supply to the distal radius and the capsule of distal radioulnar joint. Unlike MIPO techniques, we preserve the PQ muscle with the concept of minimal invasive surgery during conventional approach.

In a cadaver study by Takada et al. [7], the anatomic pictures of the pronator quadratus muscle were demonstrated. The average width from the proximal edge to the distal edge of the PQ muscle was 35.4 mm (range, 31–39 mm). Our clinical study showed this live tissue value was 37.2 mm (range, 30–41 mm). The distance from the distal edge of the PQ muscle to the lunate fossa of the distal radius was 16.2 mm (range, 12–19 mm), and the average distance from the distal edge of the PQ muscle to the scaphoid fossa of the distal radius was 17.6 mm (range, 11–21 mm). The average distance from the fracture site s to the lunate fossa of the distal radius was 12.2 mm (range, 7.3-17 mm), and the fracture site to the scaphoid fossa of the distal radius was 13.2 mm (range, 9.4-18.8 mm).

All but nine of the factures located distal to the distal edge of the PQ muscle but all were around it. In this study, 3.5 mm 3/3 T-plate (AO, Switzerland) instead of locking plate was used. The distance between the distal end of the T-plate to the center of screw hole of longitudinal limb is 15 mm, 26 mm, 43 mm for the distal, middle, and proximal hole respectively. The distance between the distal end of T-plate and the hole of the transverse limb of T-plate is 5 mm, so the plate surely could be placed over the distal fracture fragment well. Usually traction of PQ muscle could provide enough space to introduce distal two screws of the longitudinal limb of T-plate. Takada et al. emphasized that the length of the plate should be more than 52 mm to prevent damage to the PQ muscle. The proximal screw of the plate (3 holes for longitudinal limb) is usually under the PQ muscle. Inserted through PQ with blunt dissection in our study could lead less damage to the muscle. Another advantage of the sparing of PQ muscle is saving the operation time to repair as well. To simplify the measurement, AO 23-A2 fracture type was chosen in this study. This PQ muscle sparing surgery is feasible and could be provided to other type of fracture.

## Conclusion

This PQ muscle sparing surgery is feasible and can be performed without difficulty. The data might provide a useful basis for the preservation of pronator quadratus muscle applied to a functionally reduced fracture regarding the potential efficacy of conventional volar plate osteosynthesis.

## Competing interests

The authors declare that they have no competing interests.

## Authors’ contributions

HYL conceived of the study, participated in its design and helped to draft the manuscript. HYC participated in the design of the study, in the data collection process and performed the statistical analysis. Both authors read and approved the final manuscript.

## Pre-publication history

The pre-publication history for this paper can be accessed here:

http://www.biomedcentral.com/1471-2474/15/136/prepub
